# The relationships between *κ*-casein (CSN3) gene polymorphism and some performance traits in Simmental cattle

**DOI:** 10.5194/aab-65-129-2022

**Published:** 2022-03-28

**Authors:** Hamiye Ünal, Sinan Kopuzlu

**Affiliations:** 1 Animal Science Department, Institute of Science, Atatürk University, Erzurum, Turkey; 2 Department of Animal Science, Faculty of Agriculture, Atatürk University, Erzurum, Turkey

## Abstract

In this study we aim to examine genotypic structures in terms of the κ -casein (CSN3) gene locus in 70 head of Simmental cattle raised in a private enterprise in Erzurum to determine the distribution of genotypes and allele frequencies in cattle in terms of related genes and to correlate the determined genotypes with some performance characteristics. CSN3/HinfI gene polymorphisms were identified in DNA isolated from blood samples taken from Simmental cattle used in the study using the polymerase chain reaction–restriction fragment length polymorphism (PCR-RFLP) method. The frequency of the AA, AB, and BB genotype of the CSN3 gene in the population was 57.14 %, 32.86 %, and 10.00 %, respectively; the frequency of the A allele was 0.74; and the frequency of the B allele was 0.26. The mean value for the AA, AB, and BB genotypes was 5151 ± 308.6, 5805 ± 370.3, and 5772 ± 547.3 kg for real milk yield, respectively; 5313 ± 233.9, 5784 ± 280.7, and 6458 ± 414.8 kg in 305 d for milk yield; 17.9 ± 0.75, 18.6 ± 0.89, and 19.6 ± 1.32 kg for daily milk yield; and 294 ± 13.7, 316 ± 16.5, and 294 ± 24.4 d during the lactation period. The distribution of the CSN3 gene locus in the studied population is in genetic equilibrium according to the Hardy–Weinberg principle. The genotype and allele frequencies determined in terms of CSN3 gene polymorphism can be considered sufficient to reveal the genotype diversity of the breed, and it was determined that the relationship of CSN3 genotypes was only significant with 305 d milk yield ( P<0.05 ) in terms of the association between CSN3 genotypes and some performance traits. It has been concluded that animals with the CSN3 BB genotype have an economic advantage within the herd and that CSN3 can be used for marker-assisted selection (MAS) in this regard.

## 1 Introduction

While the world population was 2.5 billion in 1950, it reached 7.8 billion in 2020 (Anonymous, 2018; TUİK, 2021). Due to this increase in the world population, more food needs to be produced to feed humanity (Akman, 2012). The increase in the population requires meeting increased food needs and especially the need for animal protein. This is possible with the development of the livestock sector in every aspect. Cattle assets and yield potential, which have an important place in the livestock sector, are especially important in meeting this need. Dairy cattle breeding has a separate and important place within the livestock sector, and approximately 93 % of milk production in the world is obtained from cattle (Bolacalı, 2020).

In Turkey, one of the most important areas in the agricultural sector is animal production, and within this sector a great deal of importance is put on cattle breeding. Thus, the total number of cattle reared according to the statistics for 2021 in Turkey is 18 124 106 head (TUİK, 2021). According to Turkey's e-breeding database, the rates of Holstein, Simmental, Brown Swiss, native breeds, and other breeds registered in 2019 are 38.93 %, 36.11 %, 16.91 %, 4.84 %, and 3.21 %, respectively (Harmandar, 2019).

When the yield potential of the Simmental breed is evaluated, with its high milk and offspring yield, good fattening performance, and disease resistance, these features combine to make it preferred by producers. In Turkey, to develop livestock, culture breed animals including Simmental cattle have been brought into the country since 1925 (Koç, 2016; Akman et al., 1990). The regions where Simmental animals are most heavily bred are the central Black Sea (including Amasya and Çorum), central Anatolia (especially Afyon), Aegean, and the eastern Anatolia regions. The region with the least breeding is the Mediterranean region (Özhan et al., 2007).

Many environmental factors, such as feeding, infrastructure, and the improvement of the genetic structure of the cattle population as a result of animal import and crossbreeding studies, affect the increase in yield in cattle breeding in Turkey (Hodoğlugil, 1996; Erdem, 1997). The yield obtained from animals occurs in relation to the joint effect of phenotype, environment, and genotype. Therefore, improving both the environment and the genotype leads to an increase in yield. The phenotypic value in these quantitative traits often does not reflect the genotypic value because the characters of various yields (such as milk, fleece, egg, and meat) obtained from farm animals are under the control of a large number of genes and are greatly affected by various environmental factors. For these reasons, it is of great importance to estimate the phenotypic level of such characters (Özdemir, 2001).

If we are able reveal the existence of a sufficient degree of relationship between economic characters and polymorphic systems, it will be possible to benefit from this in the selection process by accepting the gene that determines the polymorphic characters as marker genes (Haenlein et al., 1987; Özbeyaz, 1991). Various molecular genetic methods developed in recent years are widely used in studies aimed at determining the genetic basis of milk protein systems (Formaggioni et al., 1999). Some studies have focused on the genetic polymorphisms of casein from milk proteins because of its direct relationship with milk quality, composition, and technological properties (Ceriotti et al., 2004; Dogru and Ozdemir, 2002). Studies have intensified on casein proteins, which make up 80 % of milk, and the existence of various relationships between milk yield and milk components with biochemical genetic variation detected in terms of milk protein loci such as CSN3 in recent studies (Martin et al., 2002).

This study's aim is to investigate the polymorphism of Simmental cattle raised on a private farm in terms of CSN3 gene locus from milk proteins by using the polymerase chain reaction–restriction fragment length polymorphism (PCR-RFLP) method and to reveal the distribution of genotype and allele frequencies of animals in terms of related gene location. In addition, this study seeks to investigate whether the differences in the relationship between CSN3 genotypes and some performance traits is important or not.

## 2 Material and method

### 2.1 Material

The material of the study consisted of DNA samples obtained from 70 Simmental cattle over six different lactations that were raised intensively in a private farm in Erzurum, and various milk yield records saved on farms of these animals were used to correlate the κ -casein (CSN3) gene polymorphism with milk yield.

### 2.2 Method

The genotypic structure of the κ -casein gene locus in Simmental cattle was determined using the PCR-RFLP method. Genomic DNA isolation was obtained by applying the orbitals determined in the method using the Qiagen DNA isolation kit (Purgene DNA kit, Gentra Systems, Minnesota, USA). Qualitative and quantitative controls on the obtained DNA samples were determined by using Nanodrop (Drop Plate, cat. no. 12391) spectrophotometry device.

In the PCR, the 351 bp DNA region was amplified using primers F 5 ${}^{\prime }$ -ATTTATGGCCATTCCACCAA-3 ${}^{\prime }$ and R 5 ${}^{\prime }$ -ATTAGCCCATTTCGCCTTCT-3 ${}^{\prime }$ (Doğru et al., 2008). The CSN3-Hinf1 PCR amplification protocol used was as follows: 2 µ L of each primer, 2 µ L dNTP mix (D7595: Sigma, St. Louis, MO, USA), 0.5 U of Taq DNA polymerase (D1806: Sigma), 100–200 ng genomic DNA, 3 µ L of 10× PCR Buffer (cat. no. P2192), and 1 µ L of 25 mM MgCl ${}_{2}$ . The total volume of the amplification, which is 25 µ L, is filled out with ddH ${}_{2}$ O. PCR was performed under the following conditions: 95 ${}^{\circ }$ C for 5 min, followed by 30 cycles of 95 ${}^{\circ }$ C for 1 min and 57 ${}^{\circ }$ C for 1 s, and a final extension step of 72 ${}^{\circ }$ C for 5 min.

A total of 9 µ L of each amplified sample was taken. These were put into 0.2 mL sterile Eppendorf tubes. After adding 5 µ L of Hinf1 RE enzyme (10 unit µ L ${}^{-1}$ ), the tubes were incubated at 37 ${}^{\circ }$ C for 12 h in the incubator. After cutting the DNA samples with HinfI, samples were run in a 2.8 % agarose gel, and electrophoresis was applied at 35 W for 180 min. The electrophoresis-treated gel was taken and examined under UV light.

### 2.3 Statistical analysis

Allele gene frequencies were calculated by counting each sample separately. A Hardy–Weinberg genetic equilibrium test and a chi-square independence test ( $\chi^{2}$ ) were performed to determine whether the genotype frequencies were in genetic equilibrium. The association of birth weight data of the population with genotypes was evaluated by analysis of variance using the SPSS 20.0 (Ghozali, 2009) software program. Milk yield characteristics such as real milk yield, 305 d milk yield, lactation period, and daily milk yield were investigated. Factors such as lactation period and genotype were emphasized in these yield characteristics. The following statistical model was used according to the yield characteristics in the study.

$$Y_{ijkl}:\mu +a_{i}+b_{j}+c_{k}+e_{ijkl}$$

$Y_{ijkl}$ is the value of any Simmental cow in terms of any of the characteristics of performance (real milk yield, 305 d milk yield, lactation period, and daily milk yield) considered, μ gives the population mean, $a_{i}$ is i th genotype effect (AA, AB, and BB), $b_{j}$ is the j th effect of the lactation order (second, third, fourth, fifth, sixth, and seventh), $c_{k}$ is the k th effect of calving season (winter–spring and summer–autumn), and $e_{ijkl}$ is the random error.

## 3 Result

### 3.1 Observing the PCR results

**Figure 1:**
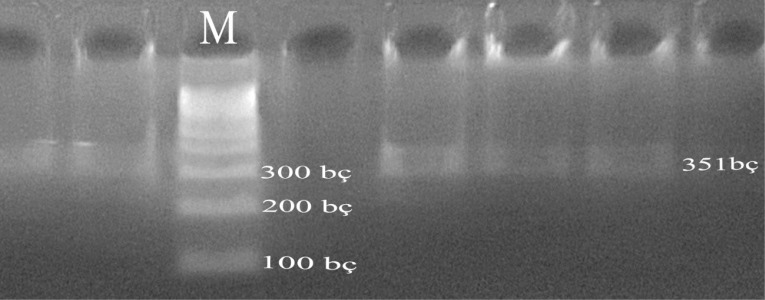
Agarose gel view of PCR products (M stands for marker; CSN3 is 351 bp).

PCR experiments were performed on each of the DNA samples obtained from the blood of Simmental cattle and on a 1.2 % agarose gel, and DNA bands were obtained (Fig. 1).

CSN3/HinfI gene polymorphic regions were determined according to genotypes BB (261/89 bp), AA (131/89 bp), and AB (262/131/89 bp).

**Figure 2:**
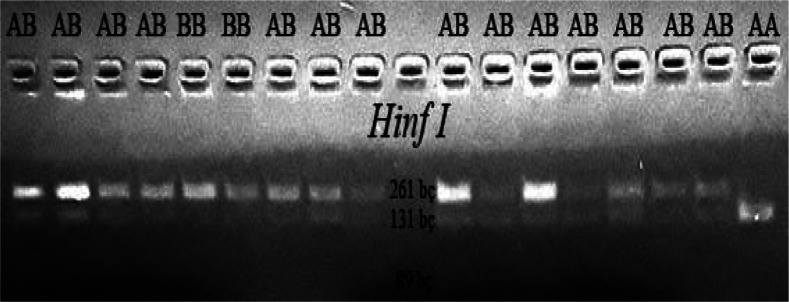
PCR-RFLP cut regions of the CSN3 gene: BB (261/89 bp), AA (131/89 bp) AB (261/131/89 bp).

### 3.2 Hardy–Weinberg genetic equilibrium test

A genotype frequency, Hardy–Weinberg genetic equilibrium, and independence test were performed, and the results obtained are presented in Table 1.

**Table 1:** CSN3 genotype frequencies and Hardy–Weinberg genetic equilibrium test results.

N	Observed			% frequency			$\chi^{2}$ test
	AA	AB	BB	AA	AB	BB	
70	40	23	7	57.14	32.86	10.00	0.1945 NS

NS means that the result was not significant at the P>0.05 level.

The genetic equilibrium of the analyzed population was evaluated based on the $\chi^{2}$ test. In the population included in the study, the differences in frequencies of genotypes for the CSN3 gene were not significant. According to the results of this study obtained for the CSN3 gene, the AA genotype had the highest genotype frequency compared to the other genotypes. Research on a population of Holstein cows estimated the frequencies of the κ -casein genotypes AA, AB, and BB to be 71 %, 26 %, and 6 %, respectively (Sitkowska et al., 2008). In another study with Girolanda cattle, the frequencies of these genotypes were determined as 54 %, 42 %, and 4 %, respectively (Barbosa et al., 2019). In both studies, the AA genotype had the highest frequency value among the genotypes, as is the case in our study, and the results of these two studies are in agreement with the results of our study.

### 3.3 Real milk yield

The overall average milk yield of Simmental cattle was determined to be 5576 ± 242.0 kg. As a result of the obtained evaluations, the highest and the lowest average values among CSN3 genotypes in terms of real milk yield were determined to result from the AB genotype (5805 ± 370.3 kg) and AA genotype (5151 ± 308.6 kg), respectively. When the variance analysis table was examined (Table 2), it was observed that the effects of genotype, lactation period, and calving season on real milk yield were observed to be insignificant.

### 3.4 The 305 d milk yield

The CSN3 genotype, which is thought to be effective in terms of the 305 d milk yield obtained from Simmental cows, was analyzed according to the factors of lactation period and calving season. The difference between the 305 d milk yield averages was found to be significant ( P<0.05 ). On the other hand, the effect of lactation period and calving season was found to be insignificant.

**Table 2:** Averages and standard error (kg) in terms of actual milk yield, 305 d milk yield, lactation period, and daily milk yield traits.

Traits		N	Real milk yield		305 d milk yield		Lactation period		Daily milk yield	
			$\bar{X}\pm S\bar{x}$		$\bar{X}\pm S\bar{x}$		$\bar{X}\pm S\bar{x}$		$\bar{X}\pm S\bar{x}$	
Overall		90	5576 ± 242		5852 ± 184		301 ± 11		18.7 ± 0.6	
*CSN3*	AA	49	5151 ± 309	NS	5313 ± 234 ${}^{b}$	*	294 ± 14	NS	17.9 ± 0.8	NS
genotype	AB	29	5805 ± 370		5784 ± 281 ${}^{\mathrm{ab}}$		316 ± 17		18.6 ± 0.9	
	BB	12	5772 ± 547		6458 ± 415 ${}^{a}$		294 ± 24		19.6 ± 1.3	
Lactation	second	9	5063 ± 650	NS	5406 ± 493	NS	292 ± 29	NS	17.5 ± 1.6	NS
order	third	17	5739 ± 48		6046 ± 365		288 ± 22		19.8 ± 1.2	
	fourth	19	5820 ± 469		5817 ± 355		328 ± 20		18.2 ± 1.1	
	fifth	21	5222 ± 434		6114 ± 329		269 ± 19		19.1 ± 1.1	
	sixth	16	5187 ± 489		5330 ± 371		309 ± 22		17.0 ± 1.2	
	seventh	8	6426 ± 682		6399 ± 517		321 ± 30		20.4 ± 1.7	
Calving	Winter–spring	53	5748 ± 335	NS	5965 ± 254	NS	299 ± 15	NS	19.3 ± 0.8	NS
Season	Summer–autumn	37	5405 ± 360		5739 ± 273		304 ± 16		18.0 ± 0.9	

NS stands for not significant at the ${}^{*}$ P<0.05 level. The difference between the averages of those denoted by the same letter (a or b) is insignificant, and the difference between the averages of those denoted by different letters is significant.

### 3.5 Lactation period

The overall average lactation period was determined to be 301 ± 10.8 d in the breed studied. When the genotypes are examined for this trait, the AB genotype has the highest overall average with 316 ± 16.5 d. The overall averages of the AA (294 ± 13.7 d) and BB (294 ± 24.4 d) genotypes were found to be close to each other. The differences between the overall averages of the genotypes, lactation period, and calving season were insignificant ( P>0.05 ).

### 3.6 Daily milk yield

The overall average value of daily milk yields in the herd studied was 18.7±0.6 kg. The overall averages of the AA, AB, and BB genotypes of CSN3 were found to be 17.9 ± 0.8, 18.6 ± 0.9, and 19.6 ± 1.3 kg, respectively. As a result of statistical analysis, the difference in daily average milk yield between genotypes, lactation order, and calving seasons was found to be insignificant ( P>0.05 ).

## 4 Discussion

The distribution of genotype frequencies of 70 Simmental cows was determined to be in equilibrium ( P>0.05 ) according to a Hardy–Weinberg genetic equilibrium test. In previous studies carried out with the same breed, the Hardy–Weinberg genetic equilibrium test results reported by researchers such as Seibert et al. (1987), Akyüz et al. (2013), and Akyüz and Çınar (2014) are consistent with the results we found.

The highest overall average value in terms of real milk yield was found in the CSN3 AB genotype (5805 ± 370.3 kg). The overall average value obtained from AB genotype cattle was determined to be 654 and 33 kg higher than the value obtained from AA and BB genotype cattle, respectively. In the study carried out by Awad et al. (2016), it was stated that the milk yield (10 726 kg) determined for the AB genotype obtained from Holstein cattle had the highest yield compared to other genotypes. This result was found to be agree with the result of this study. According to the variance analysis results, the effect of genotype on real milk yield was determined to be insignificant. The result was similar to the study conducted by Gürcan (2001), Özdemir and Doğru (2005), and Demirel (2019) in Brown Swiss and Holstein cattle. In this study, the lowest average value in terms of this trait was found in the CSN3 AA genotype, and this result is similar to the study conducted by Özdemir and Doğru (2005) on Holstein cattle.

When the data of the 305 d milk yield trait are examined (Table 2), the highest overall average was obtained in the BB genotype (6458 ± 414.8 kg), while the lowest overall average was obtained from the AA genotype (5313 ± 233.9 kg). This study showed that the difference between genotypes was statistically significant ( P<0.05 ). This result was in agreement with the finding of Soyudal (2017) in Holstein cattle. However, in contrast to our study, they were found to be insignificant in Holstein and Simmental (Kaygısız and Doğan, 1999; Demirel, 2019). Because the 305 d overall average milk yield of the cattle with CSN3 BB genotype is higher than those of the other two genotypes, the cattle with BB genotype should be multiplied in the herd.

The lactation period in CSN3 genotypes was found to be 294 ± 13.7 d in the AA genotype, 294 ± 24.4 d in the BB genotype, and 316 ± 16.5 d in the AB genotype. Among the CSN3 genotypes, the AB genotype, which has the highest overall average lactation period, was determined 25 and 30 d longer than the AA and BB genotypes, respectively. The differences between the averages of the genotypes for these traits were insignificant ( P>0.05 ). The result agrees with those reported by Doğru and Dayıoğlu (1996) in Brown Swiss, Holstein, and Simmental cattle and by Gürcan (2001) in Holstein cattle, but the opposite result was reported by Kaygısız and Doğan (1999) in Holstein cattle.

In the studies conducted by Kaygısız and Doğan (1999), Gürcan (2001), and Demirel (2019) with Holstein cattle, they associated the CSN3 genotypes with daily milk yield. When their results were evaluated in terms of the relationships between CSN3 genotypes and milk yield, it was determined that they are in harmony with the results of this study.

## 5 Conclusion

CSN3 genotypes of each individual were determined using the PCR-RFLP method from blood samples taken from Simmental breeds. It has been concluded that the genotype and allele frequencies determined in terms of CSN3 gene polymorphism can be considered sufficient to reveal the genotype diversity of the breed, and as a result of the association, the relation between the CSN3 genotypes and performance traits, which is emphasized only with 305 d milk yield, is significant. It is thought that demonstrating the usability of the determined polymorphism in animal breeding by conducting such studies and working with larger populations may provide new opportunities for defining and developing the country's livestock.

## Data Availability

The data sets are available upon request from the corresponding author.
